# Operation for the Correction of Projecting Ears

**Published:** 1910-05

**Authors:** 


### Operation For the Correction of Projecting Ears.

Ruttin (Wiener Klin. Wochensch.) makes this statement that he has not obtained satisfactory results from simple excision of the skin of the back of the ear and linear suture. Although excision of cartilage may be necessary in some cases it can usually be avoided. It has the disadvantage that injury of cartilage may be followed by perichondritis and resulting deformity. The method advised by the writer consists in first determining, by placing the auricle against the side of the head, how much skin would be required to cover the posterior portion of the auricle after the ear has been fixed in a correct position. This having been outlined, a bow-shaped incision is then made about 1 cm. behind the marked line, taking in the length of the auricle. Parallel with the first incision another curved cut is made and the portion of skin between them excised. The skin on the back of the auricle about the second incision for a width of about 1 cm. (if necessary, as far as the helix) is then mobilized. The margins of the two cuts are then approximated. To secure accurate apposition this may be supplemented by excision of wedge shaped portions of skin. A compress bandage is applied and allowed to remain for fourteen days until superficial adhesion has occurred.—*Charlotte Medical Journal.*

